# Hybrid global search with enhanced INC MPPT under partial shading condition

**DOI:** 10.1038/s41598-023-49528-w

**Published:** 2023-12-14

**Authors:** Mohamed Zaki, Ahmed Shahin, Saad Eskender, Mohamed A. Elsayes

**Affiliations:** Electrical Engineering Department, Faculty of Engineering, Mansoura University, Mansoura, Egypt

**Keywords:** Electrical and electronic engineering, Energy infrastructure

## Abstract

The photovoltaic system is quickly emerging as a highly favored option among renewable energy sources. However, it faces several significant challenges, including variable solar irradiance, temperature, and partial shading. Unfortunately, conventional Maximum Power Point Trackers (MPPTs) cannot accurately track partial shading. Artificial intelligence and optimization techniques have been proposed as alternatives, but they require extensive training and can take a long time to achieve maximum power point (MPP) under partial shading circumstances. In this paper, a dynamic and fast-moving method of MPP tracking is proposed for use under both uniform solar irradiance and partial shade. This method combines an enhanced incremental conductance (INC) algorithm with a global search algorithm that looks at how well solar cells work when partly shaded. Simulation investigations are performed to validate the method's applicability and ensure that it reaches the most accurate value of MPP with a short-tracking time of less than 0.2 s and a steady-state error of less than 0.3% of the PV power. The results confirm the efficacy of the suggested tracking method under uniform solar irradiance and partial shade.

## Introduction

Solar energy is a highly advantageous form of energy, as it is both cost-free and safe and has a high energy output. Additionally, it is environmentally friendly as it produces no greenhouse gases and is derived from the abundant and renewable energy source of the sun, which provides 1,000 times more daily energy transfer to Earth than fossil fuels. Even though photovoltaic (PV) systems have these benefits, their nonlinear V–I characteristic, which is affected by things like solar irradiance and temperature, makes it hard for them to be widely used for energy production^1,2^. N-type and P-type semiconductors make up PV cells. The N-type is in the cell's front, whereas the P-type is at the rear^3,4^. Expanding PV array production can increase the overall efficiency of solar panels, resulting in a higher maximum energy rating for the system^5^.

The strength of the irradiance influences the photogenerated current. At a constant temperature, the intensity of irradiance over a broad operational range directly correlates with the short-circuit current of a solar cell, whereas the open-circuit voltage is only slightly proportional. Temperature variation also influences both the I–V and P–V characteristics of a PV cell. A rise in temperature causes a reduction in open-circuit voltage while the short-circuit current increases. Band theory, which originates in solid-state physics, is used to characterize this behavior. The Fermi energy level shifts closer to the middle of the forbidden gap as temperature increases. Both modifications reduce the photovoltaic voltage by lowering the potential barrier in the lighted P–N junction^6,7^.

MPPT ensures the PV system constantly operates at peak power by varying the PV string load. MPPT controllers employ specialized algorithms to continuously regulate the panel operating voltage to gather the PV panel's maximum power^8^. Many conventional MPPT approaches for solar PV panels have been published. The short-circuit current method, open-circuit voltage method, Perturb and Observe (P&O) methods^9,10^, INC methods^5^, fuzzy logic, and metaheuristic methods are examples of MPPT techniques^11–15^. These approaches differ from one another in terms of their usability, rate of convergence, system dependability, and ability to effectively track MPPs. The primary concerns for solar PV array MPP tracking are (1) reaching MPP quickly, (2) stabilizing at MPP, (3) seamlessly transitioning between MPPs as the weather changes, and (4) obtaining the GMPP under partial shade conditions. Overall, solar PV power production requires a fast and dependable MPPT^16^.

Solar PV panels are regularly arranged in series and parallel in large-scale photovoltaic systems. When a shadow covers a particular solar PV panel, the result is a hot spot, distorted I–V characteristics, and decreased output power. Numerous factors can cause partial shading, including clouds, structures, trees, and dust. As well as partial shading reduces the panels' output power, multiple peaks occur in the series-connected modules^17^. Connected solar cells of diverse types or functioning under different conditions produce a mismatch. The mismatch is the main source of PV system energy loss^18^. Under partial shading, shaded PV cells absorb energy from unshaded cells resulting in highly localized power loss and hot spots. As a result, the power generated by shaded panels is significantly lower than that generated by unshaded ones. This may damage PV modules and reduce PV system performance^18^. Hence, bypass diodes are linked across solar modules to prevent shaded PV modules from absorbing electricity from unshaded ones^7,19,20^. The electricity then goes via bypass diodes when there are shaded solar panels. Using bypass diodes means that the I–V and P–V curves of the PV array might have many power peaks under non-uniform irradiance, which makes MPP tracking more difficult.

Up to 70% of a PV system's electricity might be lost due to partial shading. Power efficiency, minimal power losses, and high output power may be achieved by precisely tracking GMPP^21,22^. Conventional MPPT approaches are ineffective in distinguishing between the GMPP and LMPPs^7,19,20^. Several MPPT algorithms, such as the P&O and the INC approaches, have been recently improved for tracking the MPP. Most MPPT approaches under partial shading use artificial intelligence, neural networks, and particle swarm optimization (PSO) algorithms. However, these techniques often require longer processing times and more complicated control systems to reach the GMPP^11,12,23–27^. The GMPP cannot be detected using the classical and improved classical MPPT techniques^28,29^.

New artificial intelligence-based algorithms are being developed to track the GMPP in the presence of partial shade. One of these methods is a neural network-based algorithm, as described in^30^. The neural network technique has fast tracking speed, low steady-state oscillation, and fast irradiance response. Its limitations include relying on the PV array model for tracking accuracy and using simulated models for training data. Fuzzy logic optimization approaches are simple, rapid, dynamic, and have low steady-state oscillation^31^. Fuzzy rules can negatively impact system performance, and the selection of fuzzy parameters can also affect it.

Many new metaheuristic algorithms, such as the Bat metaheuristic algorithm with dynamically narrowing search space^32^, most valuable player algorithm^33,34^, advanced limited search strategy^35^, radial movement optimization, teaching–learning-based optimization^36^, reduced search space exploration^37^, and adaptive Jaya^14^, were proposed to track the GMPP. While these methods offer the advantages of improved efficiency, reliability, and minimal fluctuations compared to classical metaheuristic techniques, they still require intricate controllers to manage a large volume of measurements.

MPPTs based on genetic algorithms^38^ have the benefits of less complexity and the ability to operate under partial shade, but their primary disadvantage is slow tracking speed. MPPT based on the grey wolf approach is proposed in^39^. Despite the benefits of few parameters and simplicity of implementation, the grey wolf algorithm's tracking speed is slow. Several P–V curve scanning algorithms combine P&O or INC with global search to find the GMPP, as shown in^40–43^. Although these approaches capture the accurate GMPP with high tolerable accuracy, they necessitate an excessive number of search operations, which reduces tracking speed and system efficiency during extended searching times.

An examination of similar research shows that previous studies have focused on efficiently monitoring MPP while reducing fluctuations in output power, highlighting this as a clear area of concern. However, the approaches are hindered by their sluggish tracking speeds, which can be attributed to the high volume of necessary operations. Additionally, they tend to lose track of GMPP, exhibit fluctuations around MPP and result in significant power losses during the tracking process. The main objective of this research is to develop an MPPT system that overcomes the drawbacks of comparative approaches, offering the following advantages:

- Utilizing the improved INC algorithm, it swiftly reaches the MPP in less than 40 ms.
- It consistently achieves standard tracking times of under 0.2 s across all operating conditions.
- The stability of PV output power at the MPP exhibits minimal variation, only 0.3%.
- Superior power efficiency, enhanced reliability, and reduced implementation costs.

This paper combines simple classical methodologies with several improvements based on PV system output curve trend analysis under partial shade. The method proposes combining the open circuit method and INC with modifications to determine the GMPP more efficiently and with a controller that is both simple and stable. Firstly, a study of the PV panels will be conducted under various irradiance and temperature conditions on single and multiple PV panel configurations. The performance of PV panels in partial shade will also be investigated. Secondly, the proposed strategy will include three significant phases. Firstly, the number of series panels will be determined to identify the number of LMPPs. The open-circuit voltage technique will be used to estimate the locations of LMPPs. Secondly, the proposed technique will attempt to obtain MPP for all series panels (N) using the classical open circuit voltage algorithm and save it as an LMPP. Another possible LMPP will be determined using the same algorithm by taking the open circuit voltage of the (N-1) combination of series panels and so on until all required LMPPs have been stored. When comparing the PV output power of LMPPs successively, the point with more power than the two points directly preceding and following it will be close to the GMPP. Thirdly, an improved variable step INC will search for the GMPP, beginning the search from the place where the earlier global search ended. Any change in environmental conditions will be projected as a meaningful change in output power, alerting the suggested algorithm to look for a new GMPP. Lastly, a Simulink model will be used to evaluate the suggested technique in various partial shading and progressive irradiance change scenarios.

## PV panels model

The physical model of the PV cell must be examined early on to discover a solution to the partial shadow problem. Photocells provide the scientific foundation for photovoltaic cells. Photocells emit electrons when exposed to light, and their energy of motion is determined by Eq. (1)^44,45^. Equation (1) expresses the kinetic energy obtained by one electron following its release as the product of the electron charge multiplied by the voltage supplied to the photocell. While the photocell is a current source, the number of electrons released is proportional to the intensity of the incident irradiance. The voltage applied to the cell plays a crucial role in determining the kinetic energy of the electron as well as the quantity of electric energy produced by the photocell.

1 $${e}^{-}{\text{V}}=\frac{1}{2}{{(m}_{e}\cdot v)}^{2}$$

where ${{\text{e}}}^{-}$ is the electron charge, V is the cell voltage difference, ${{\text{m}}}_{{\text{e}}}$ is the electron mass, $v$ is the electron speed.

**Figure 1:**
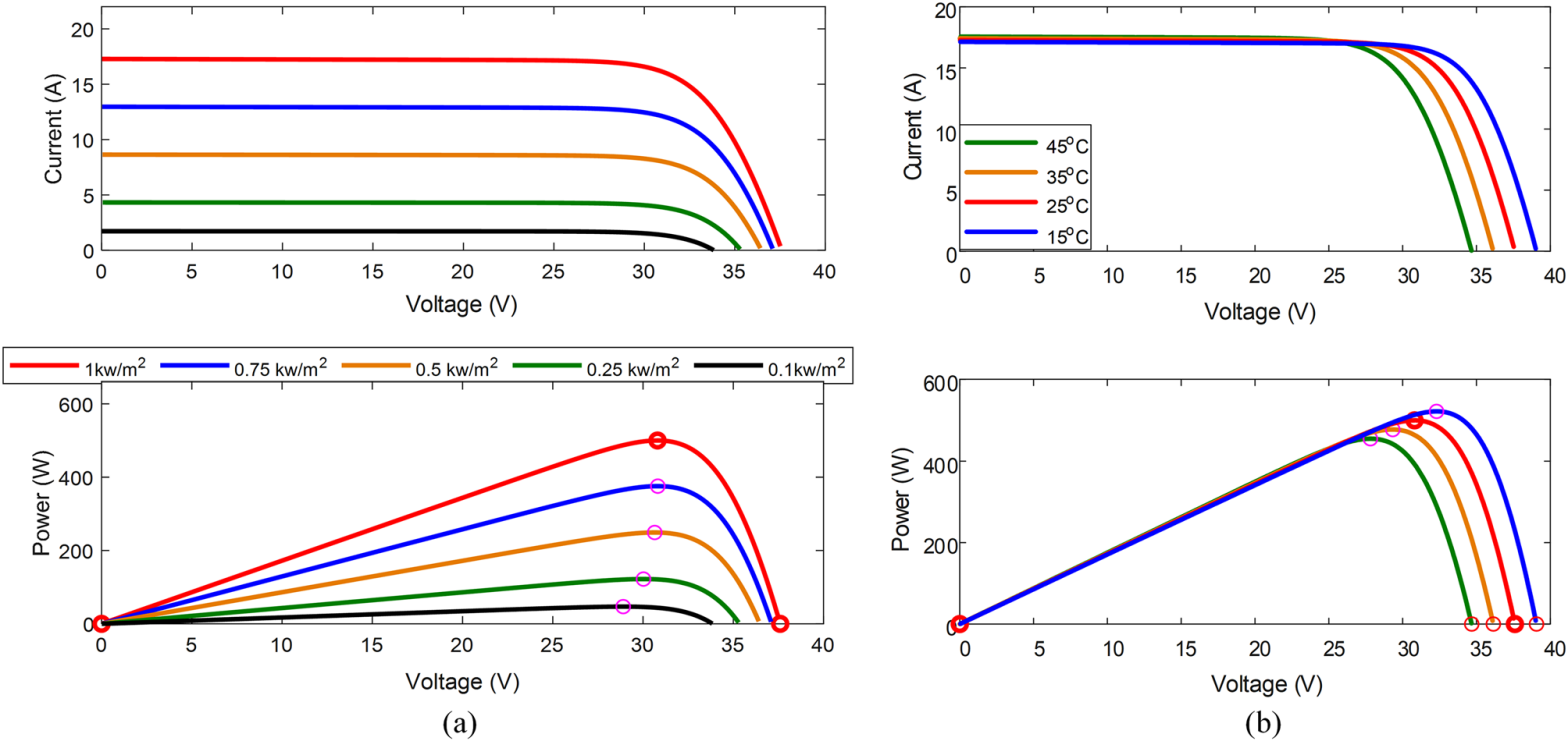
P–V and I-V characteristics of PV panel (**a**). Different irradiance (**b**). Different ambient temperatures.

PV cells are formed by connecting n-type and p-type segments to create an effective region, which is a zone of positive and negative ions that generate a potential difference. When these cells are exposed to light, electrons are released and flow through the electrical circuit connected to the cells under the influence of the effective region potential. Figure 1a demonstrates the performance of a solar panel. As the cell's voltage decreases, the current increases rapidly until it stabilizes. At a certain point, the current becomes constant, which is the maximum current of the cell. Similar characteristics are observed when the irradiance intensity is varied. In this case, the current is proportional to the irradiance intensity, and a small open-circuit voltage that is also proportional to the irradiance intensity can be observed. When examining electric power, it is found that it increases as the voltage decreases until it reaches its maximum value, after which it decreases linearly until it reaches zero. Figure 1b shows a slight increase in current and a drop in open circuit voltage as the temperature increases.

**Figure 2:**
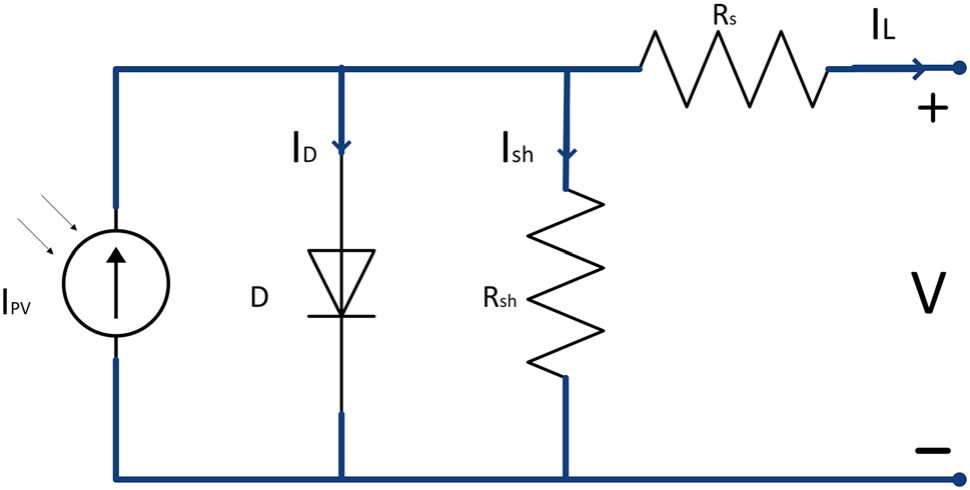
Solar cell electrical model.

In an electric circuit, a current source parallel to a diode can be used to depict an ideal solar cell. Install two resistors, as depicted in Fig. 2, to represent electrical losses. One with a low resistance in series and the other with a high resistance in parallel^46,47^. A part of the current, as in Eq. (2), is dissipated as the diode current, which is calculated by multiplying the saturation current of the diode by the natural exponential ratio of the energy acquired by the electrons by the diode's energy scale factor. The shunt resistance causes a small loss of current. A portion of the energy is also lost due to the series resistance, reducing the cell voltage.

2 $${I}_{L}={I}_{PV}-{I}_{D}-{I}_{sh}={I}_{PV}-{I}_{o}\left({e}^{\left(\frac{{e}^{-}{\text{V}}}{\alpha KT}\right)}-1\right)-{I}_{sh}$$

where I_L_ is the load current, I_PV_ is the PV cell current, I_O_ is the diode saturation current, I_sh_ is the shunt resistor current, T PV temperature in kelvin, K Boltzmann constant, α diode ideality factor, IPV represents the PV current, I_D_ is the diode current, R_sh_ is the shunt losses, and Rs is the series resistance of panel^19^.

### PV panels under partial shade

Like other energy sources, solar cells can be linked in parallel and series to generate high current and voltage outputs. Under typical temperature and irradiance conditions, solar panels behave like a single cell, with the voltage doubling in series connection, the current doubling in parallel connection, and the electric power doubling in both cases. However, under varying irradiance across panels, the graph depicting electric power against voltage may exhibit several peaks. Only one of these peaks represents the MPP, which is the optimal operating point for the solar panel to achieve its highest efficiency.

#### Series PV panels under partial shade

**Figure 3:**
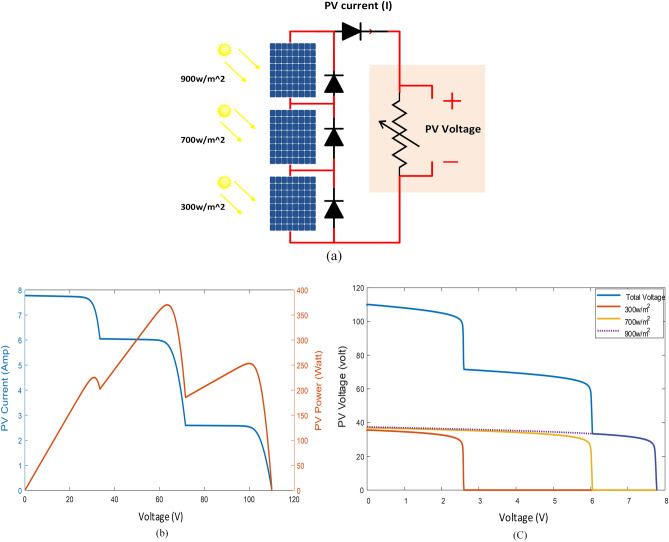
Connection of three series panels exposed to various levels of the shadow: (**a**) connection of panels, (**b**) P–V characteristics, (**c**) I-V characteristics.

Research on the P–V curve of series-connected solar panels under varying irradiance conditions is shown in Fig. 3a. The curve in Fig. 3b exhibits three peaks, with the MPP occurring at a voltage of 63 V. Since all panels have the same current, the output voltage is the sum of the individual panel voltages. This can be verified using three independent sources connected in series, dividing the voltage proportionately to the current flowing through the circuit. As the current reaches 2.6 amps, the maximum current of the panel exposed to 300 W/m^2^, the panel voltage decreases with the same current until it is disconnected as the load voltage drops.

Afterward, the current continues through the parallel-connected diode of one panel while the other two panels remain in operation as the voltage drops. When the voltage drops further, the process is repeated at a current of 6.1 A with a panel exposed to 700 W/m^2^. Figure 3c illustrates the relationship between load voltage, panel voltages, and current. This graph demonstrates why the power curve has multiple peaks and how, as the voltage decreases, the currents in the panels increase to their maximum values until the panels cannot handle any additional current.

#### Parallel PV panels under partial shade

**Figure 4:**
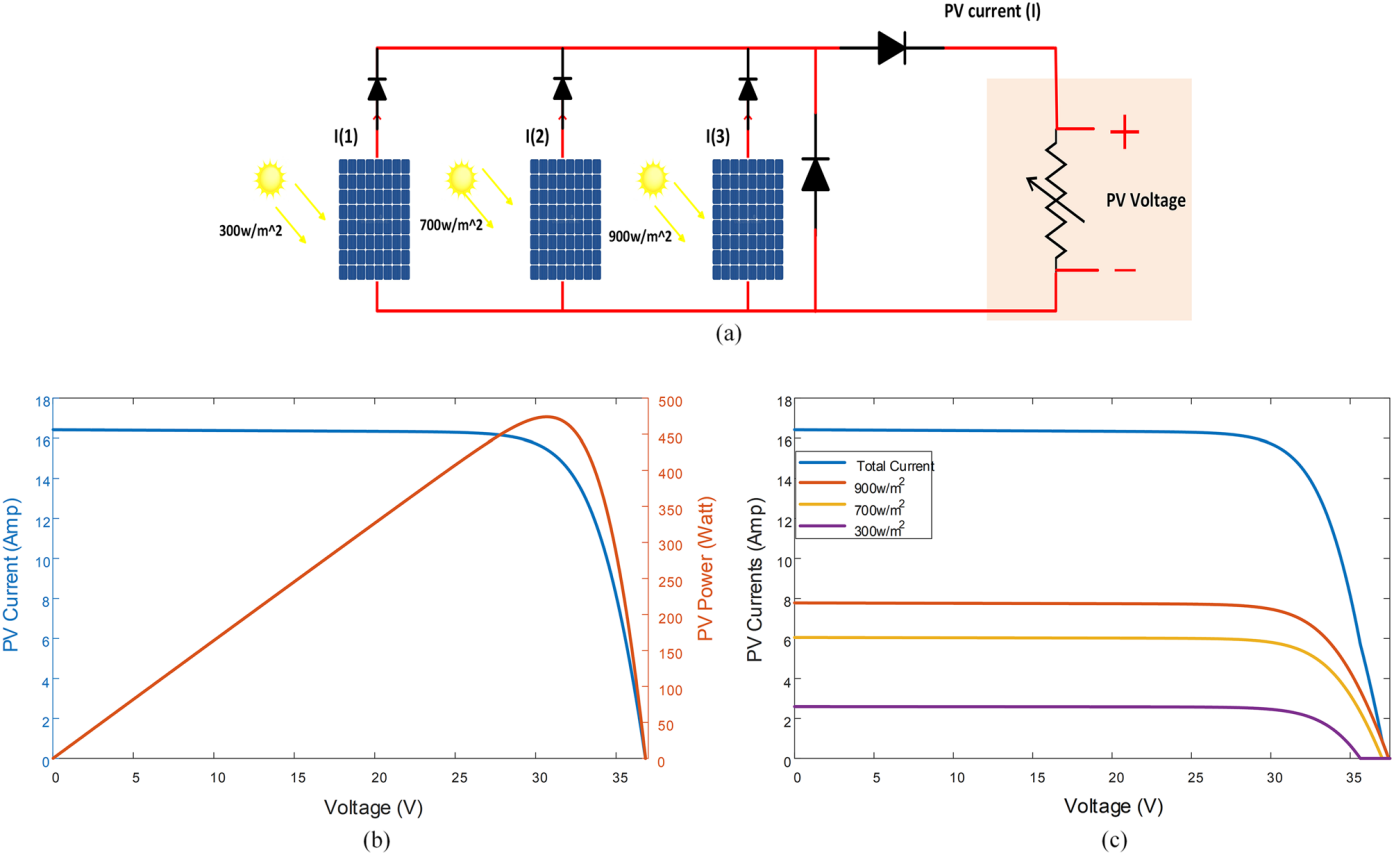
Connection of three Parallel panels exposed to various levels of the shadow: **a)** connection of panels, **b)** P–V characteristics, **c)** I-V characteristics.

Panels with the same voltage can be connected in parallel, as shown in Fig. 4a. The system's performance curve is depicted in Fig. 4b, which demonstrates that the panel group has one maximum power point (MPP) despite different irradiance levels. In Fig. 4c,a panel exposed to 900 W/m^2^ feeds the load alone at its open-circuit voltage. As the voltage drops to 37.5 V, the panel exposed to 700 W/m^2^ joins in, and each panel contributes a portion of the current that is proportional to the value of the applied voltage.

Since there is only one MPP, traditional maximum MPPT methods can easily determine this point. Comparing the panels' performance in Fig. 4b, they produce a maximum power output of 475 W, which is greater than the performance of the same panels in series, which reached only 370 W in Fig. 3b.

After examining the performance of solar panels connected in both series and parallel arrangements, parallel connections are preferable in cases of partial shading. This is because all panels contribute to power generation with values close to their maximum power, resulting in higher overall efficiency. However, due to the high voltages required to support loads, series connections are necessary despite the benefits of parallel connections. As a result, MPPT methods must include a mechanism to catch the MPP in the event of partial shading.

For best performance, panels in series should have the same current, while panels in parallel should have similar voltage characteristics. When connecting multiple panels, it is advisable to connect them in small groups in parallel before connecting these groups in series.

The previous analysis has provided several key findings that will assist in designing an MPPT system for use under partial shade conditions. Firstly, the maximum number of peaks that can appear in the P–V curve corresponds to the number of panels connected in series. However, this number can be reduced to a single peak under regular irradiance, while it varies under different partial shade scenarios. Secondly, the peaks projected onto the voltage axis are situated near the sum of the MPP voltages of the series-connected panels. The first peak represents the MPP voltage of a single panel, the second peak represents the sum of two panels' MPP voltages, and so on until all the panels' MPP voltages are reached.

Thirdly, the relationship between current strength and irradiance can be utilized to measure the amount of irradiance striking each panel group. Additionally, by determining the power value at one LMPP and the number of panels providing power at that point, it is possible to ascertain whether the next LMPP will have a greater power output.

Finally, it is important to note that the GMPP does not appear randomly between the LMPPs. Instead, it is observed that the power at the LMPPs gradually increases until it reaches the GMPP and then decreases again under partial shade conditions. Thus, it can be concluded that in all cases of irregular irradiance, the GMPP moves predictably between the LMPPs, starting close to the open circuit, where the power gradually increases and then declines again after reaching the GMPP.

## Proposed MPPT algorithm

**Figure 5:**
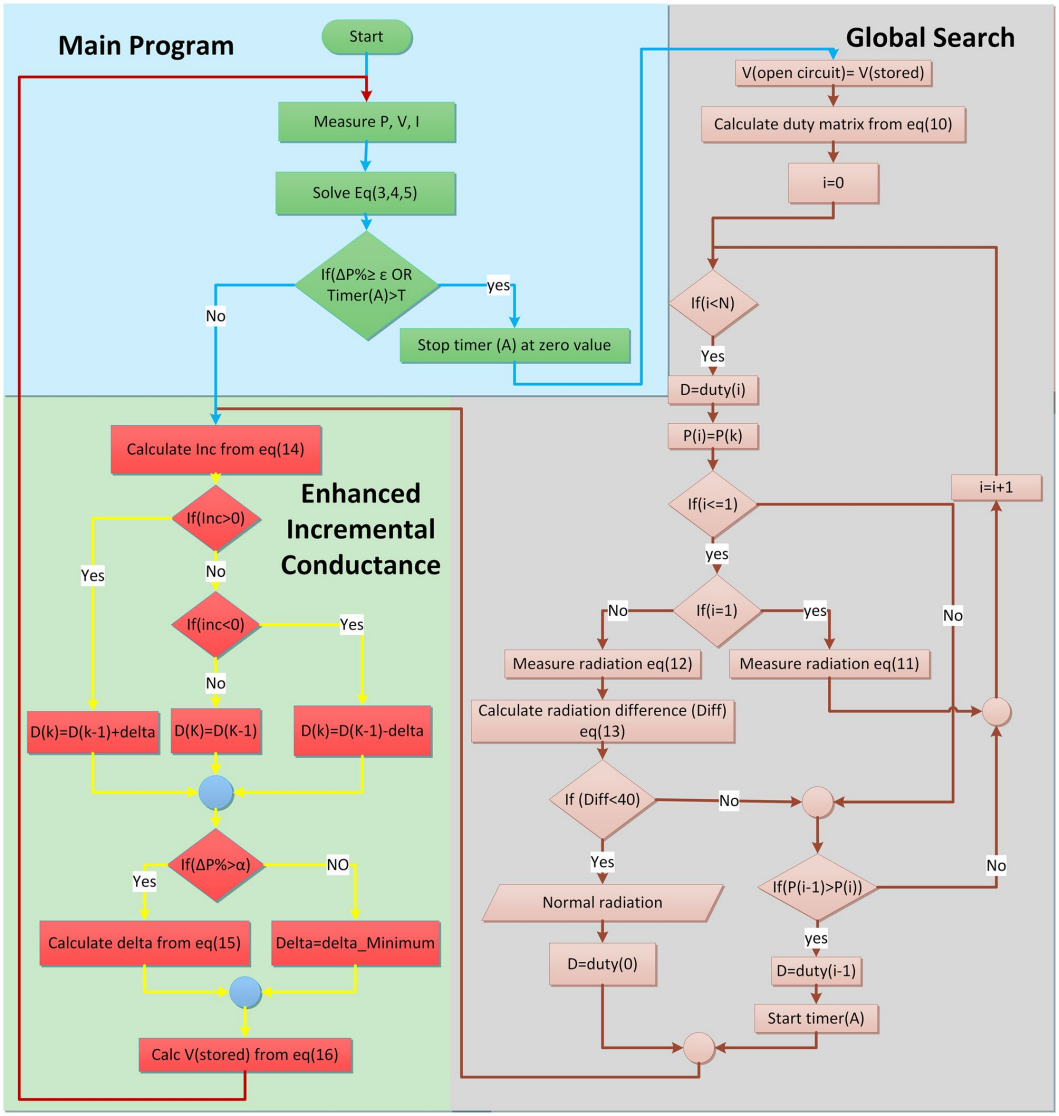
The flowchart of the proposed MPPT.

The proposed approach seeks to quickly capture the precise MPP under partial shading, normal irradiance, rapid and gradual variation in irradiance, and temperature change. Figure 5 represents this approach, which operates in two primary stages: the first, termed global search, determines a starting point in proximity to the GMPP, while the second, improved INC, identifies the precise MPP.

### Global search technique

Initially, the global search tool is employed to identify whether the phenomenon under investigation involves partial shading, normal temperature, or irradiance variation. The subsequent four phases demonstrate the global search process, which combines the open-circuit MPP tracking method with the trend of the P–V curve under partial shading.

1- The first stage is to detect a change in solar irradiance, either partially or completely, by detecting a change in output power owing to Eq. (7).

3 $$\Delta P={P}_{k}-{P}_{k-1}$$

4 $$\Delta I={I}_{k}-{I}_{k-1}$$

5 $$\Delta V={V}_{k}-{V}_{k-1}$$

6 $$\Delta P\mathrm{\%}=\frac{{P}_{k}-{P}_{k-1}}{{P}_{k-1}}$$

7 $$\left|\frac{{P}_{i}-{P}_{i-1}}{{P}_{i-1}}\right|>\varepsilon $$

where: ε is the highest allowable limit of power fluctuation without changing the irradiance and is taken to be [5%:10%] of PV system output power^23^. If the requirement of Eq. (7) is met, the global search begins.

2- The next step involves estimating the number of expected LMPPs. Each of these LMPPs can be approximated using the standard open circuit voltage ratio method in Eq. (8) by shading 1 to N panels as in Eq. (9). If the number of series panels equals N, the number of LMPPs will also be N. The proposed points will follow Eq. (9). The N-series PV panels' subsequent LMPPs can be calculated using Eq. (10).

8 $${V}_{mpp}=\left[70\%:80\%\right]{V}_{oc}$$

9 $${V}_{{mpp}_{i}}=[70:80\%]\frac{N-i}{N}{V}_{oc}$$

10 $$ {\text{duty}}\left( {\text{i}} \right) = 1 - \frac{{V_{oc} \cdot ratio\left[ {70:80{\text{\% }}} \right] \cdot \frac{N - i}{N}{ }{-}\left( {\text{i}} \right) \cdot V_{diode} }}{{V_{load} }} $$

where: i=0,1,2,3,….,N-1, i=0 represents the duty ratio of the first MPP located next to the open−circuit voltage, where all panels contribute to generating power, i=1 represents the duty ratio of the possible location of the second MPP, where only one panel is shaded and does not participate in generating power, and so on for i= 2,3,….,N-1, V_Diode_ is the voltage over the bypass diode of the shaded panel.

3- At this stage, the global search algorithm detects partial shading. Calculate the power and irradiance of the first two LMPPs starting from the right side of the P–V curve (i = 0 and i = 1), and then determine their irradiance difference. Since the irradiance directly correlates with the current of the photovoltaic cell, it is possible to indirectly measure the irradiance from the current, as demonstrated in Eqs. (11) and (12)^43^. As mentioned in Reference^48^, if the difference between the irradiance intensity at the MPP and the short circuit point is less than 40, there will be no partial shading. In the proposed system, after studying the P–V curve, it was observed that under uniform irradiance conditions, the current remains relatively constant from the short circuit point up to an area close to the MPP. Therefore, the short circuit current was substituted with the current at point $i=1$ in Eq. (11), located within that region. If there is a discrepancy in irradiance between $i$ = 0 and $i$ = 1, the system identifies partial shading and progresses to step 4. Otherwise, if no shade is detected, the controller proceeds directly to the enhanced INC stage.

11 $$irradiance(i=1)=\frac{{I}_{pv}\cdot 1000}{{I}_{sc}\cdot M}$$

12 $$irradiance(i=0)=\frac{{I}_{pv}\cdot 1000}{{I}_{mpp}\cdot M}$$

13 $$Diff=irradiance \left(i=0\right)-irradiance (i=1)$$

**Figure 6:**
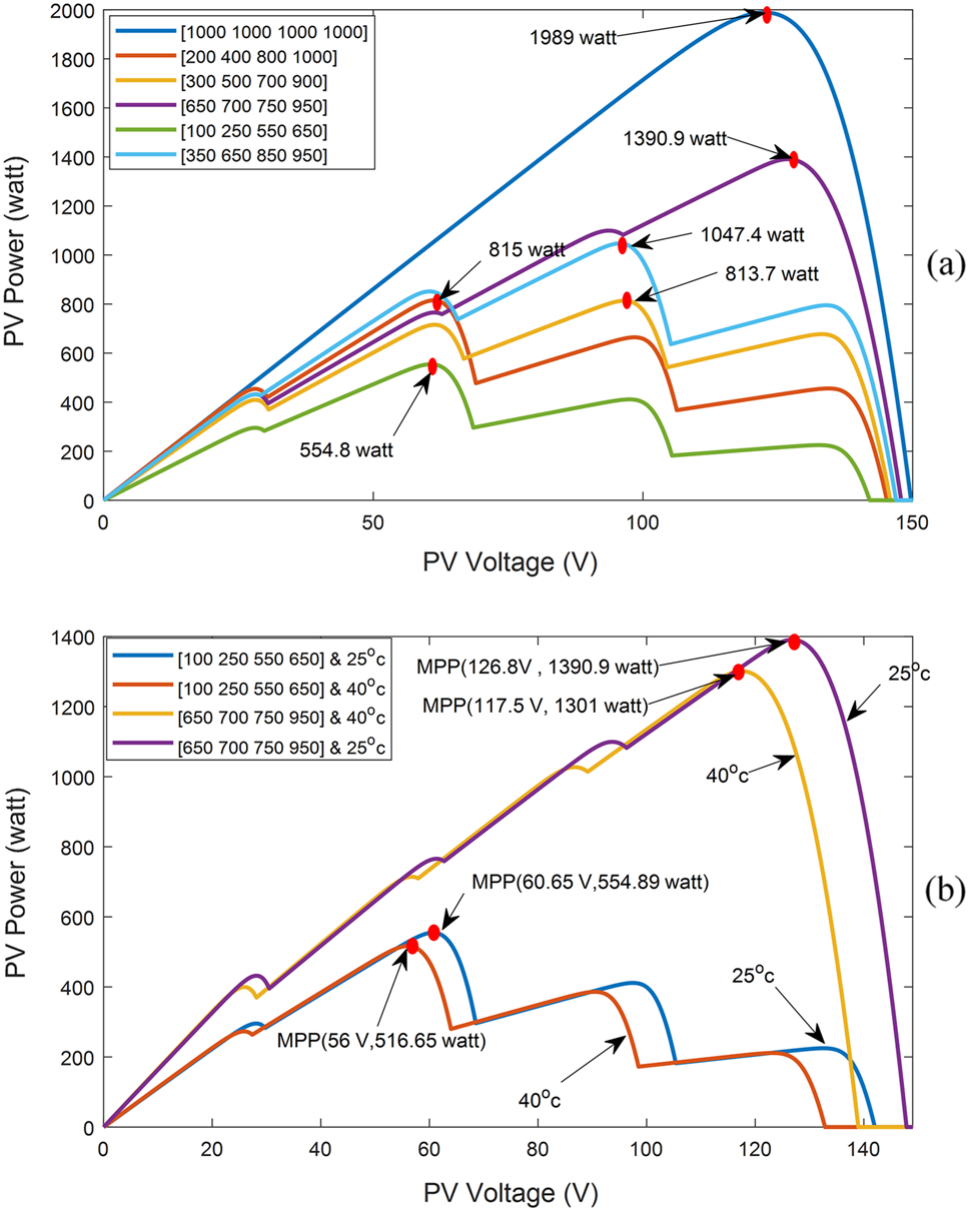
P–V Characteristics: (**a**) Under different partial shadow patterns, (**b**) In case of temperature change under partial shadow patterns.

4- In the event of partial shade detection, each LMPP has the potential to represent the GMPP. Therefore, it is necessary to examine the duty ratio of each point to determine output power. Beginning at i = 0, the suggested LMPP duty ratio is evaluated using Eq. (10). The observation is gleaned from the analysis of the P–V curve trend displayed in Fig. 6, demonstrating that the LMPPs' power steadily rises until it attains the GMPP, after which it diminishes. The output power of the first LMPP is measured, and the output power of the second LMPP is also measured. They are then compared to each other. If the power of the first LMPP is greater, it becomes the GMPP; otherwise, the power of the third LMPP is measured and compared to that of the second LMPP. If the power of the second LMPP is greater, it becomes the GMPP; otherwise, the system continues in the same way until the GMPP is determined. The next stage will begin at the LMPP with the highest power.

### Enhanced INC

To achieve a swift search and minimize steady-state error, a variable-step enhanced incremental conductance (INC) is employed. This enhanced method is introduced to counteract the sluggishness of the tiny step INC, the fluctuation of the large step INC, and the instability of the variable step INC. The proposed algorithm's operation will be shown in the four steps that follow:

1- The INC tests shown in Eq. (14) will begin from the position chosen by global search:

14 $$\left\{\begin{array}{c}error=\frac{\Delta I}{\Delta V}+\frac{I}{V}\\ If(error<0)\\ then({D}^{n}={D}^{n-1}+\Delta D)\\ ElseIf(error>0)\\ then({D}^{n}={D}^{n-1}-\Delta D)\\ else\\ {D}^{n}={D}^{n-1}\\ End\end{array}\right.$$

**Figure 7:**
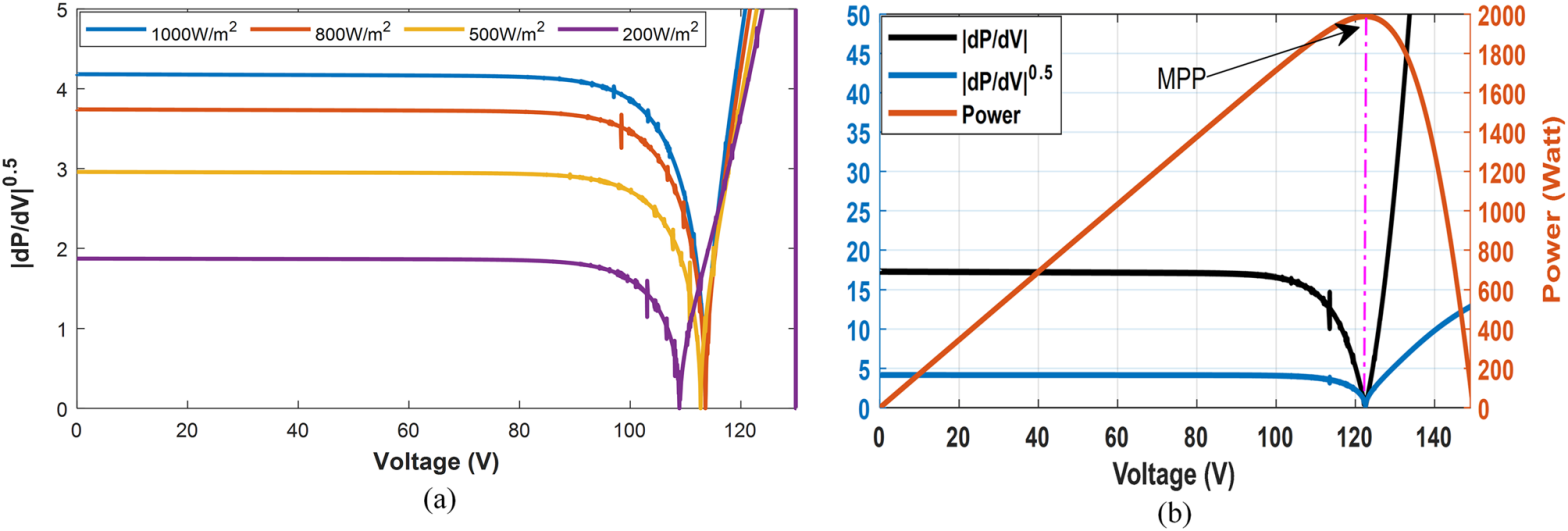
(**a**). the absolute change of √(|dP/dV|) overvoltage at different irradiance levels. (**b**). comparison between the proposed $\surd (|{\text{dP}}/{\text{dV}}|)\mathrm{and absolute }|{\text{dP}}/{\text{dV}}|\mathrm{ overvoltage}$.

2- Optimizing the variable step improves performance, speed, and minimizes steady-state error. Several methods have been proposed to determine a desirable step size^49^. Although |dP/dV| exhibits significant overshoot and moderate tracking speed, it demonstrates good dynamic performance and low steady-state error when compared to other approaches mentioned in^49^. The relationship between output power and voltage is described as a polynomial function in^50^. Based on this polynomial function, Eq. (15) performs well in reaching the MPP, displaying good stability and a fast response. The use of √(|dP/dV|) is preferable to |dP/dV| alone, as it speeds up the MPP-seeking process. Figure 7a illustrates how the performance of √(|dP/dV|) varies across different irradiance intensity levels. In Fig. 7b, it can be observed that when the system is far from MPP, both √(|dP/dV|) and |dP/dV| remain nearly constant. However, when the system is near the MPP, √(|dP/dV|) exhibits a slower decrease. The global search approach ensures that the system typically operates in a region near the MPP, making √(|dP/dV|) preferable choice. This is because the slow decrease in step size facilitates a quicker attainment of the exact MPP. The large step-size INC method fails to stabilize at the MPP and instead fluctuates around it, resulting in significant power losses. In contrast, the small step-size INC method necessitates an excessively long tracking time to reach the MPP. Conversely, the proposed method effectively combines the advantages of rapid convergence and stability at the MPP.

15 $$\Delta D=\gamma \sqrt{\frac{\Delta P}{\Delta V}}$$

where:$\gamma$ is a scaling factor.

3- To adjust for temperature and irradiance variations, the open circuit voltage is measured using Eq. (16) without disconnecting the panels, which is the inverse calculation of Eq. (9). This step is crucial as it enables the adjustment of open-circuit voltage. It is important to note that both the open circuit voltage and the MPP voltage are subject to temperature fluctuations, as visualized in Fig. 6b.

16 $$V\left(store\right)=\frac{N\cdot {V}_{GMPP}}{80\mathrm{\%}(N-i)}$$

4- If partial shading is detected, a fixed timer is initiated to reset the main algorithm to step one without the need for a condition in Eq. (7). Using a timer is crucial because, in cases of partial shadow, the current flows through a diode parallel panel with partial shade within the string. When irradiance changes on these panels, two scenarios can arise. First, if irradiance increases on these panels, they can pass current with the rest, leading to a change in total voltage and current, which the system detects. Secondly, if there is a change in irradiance intensity, it may be insufficient for these panels to contribute to the current and reset the global search. A timer with a fixed value will be programmed to reinitialize the system to detect if there is a new maximum power point.

## System configuration

**Figure 8:**
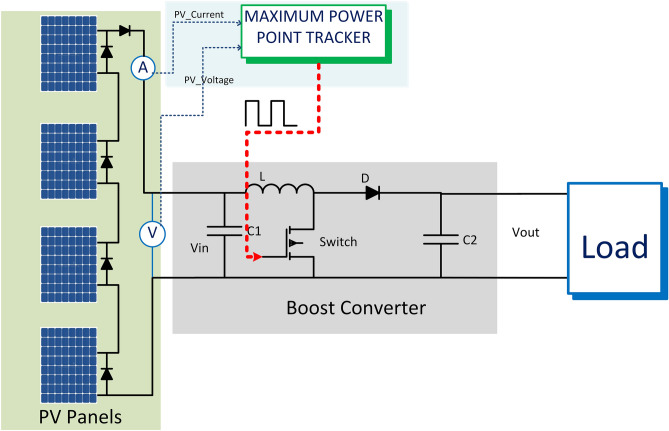
PV system block diagram.

The system depicted in Fig. 8 comprises four series of panels, a DC/DC boost converter commanded by the proposed MPPT approach, and the system's load. Each solar module is linked in anti-parallel with bypass diodes to mitigate the impact of mismatch losses from modules connected in series and prevent hotspots on the panels. When the PV modules are shaded, electricity flows through these diodes. However, a significant disadvantage of a series PV module is the substantial power loss when a single solar panel or a group of solar panels is subjected to partial shading.

Table 1 displays the characteristics of the PV panel utilized. Figure 1a presents the P–V and I-V curves of the selected module under varying irradiance conditions with a constant temperature. These curves demonstrate a rise in short-circuit current and a slight increase in open-circuit voltage, leading to an increase in output power as irradiance levels increase. In Fig. 1b, the P–V and I-V characteristics are shown to change because of temperature variation. As temperature increases, there is a small increase in current accompanied by a decrease in panel voltage, resulting in a reduction in output power and a shift in the position of the MPP.

**Table 1:** Parameter of the used PV module:

Parameter of PV module	Value	Parameters of DC/DC converter	Value
Maximum power (P_max_)	250 W	İnput capacitor	2200 uF
Maximum power point voltage (V_MPP_)	30.8 V	Output capacitor	2200 uF
Maximum power point current (I_MPP_)	8.12	İnductor	3 mH
Open circuit voltage (V_OC_)	37.6 V	Switching frequency	2500 HZ
Short circuit current (I_SC_)	8.64		

Figure 6a depicts the P–V curves of various partial shadow patterns and identifies several LMPPs but only one GMPP for each pattern. The location of LMPPs varies slightly across all patterns, with the irradiance changing at a fixed temperature, as shown by a trend analysis of the curve in Fig. 6a. Despite the curve trend results indicating that the GMPP will be the LMPP with the most power, it varies between all LMPPs with a gradual decrease from both sides. For instance, in pattern [100, 250, 550, 650], the GMPP is at 60 V, whereas in irradiance pattern [650, 700, 750, 950], it is at 124 V.

Figure 6b shows the impact of temperature changes on shade patterns or a study of partial shading at different temperatures. This figure presents the P–V curves of [650, 700, 750, 950] irradiance at two temperatures of 25 °C and 40 °C. Despite the curve trend analysis indicating that the GMPP voltage similarly decreased from 125 V at 25 °C to 117 V at 40 °C, the open circuit voltage shifted from 148 V at 25 °C to 139 V at 40 °C. Another irradiance pattern, [100 250 550 650], is tested at the same two temperatures in the same figure. In this case, the open circuit voltage shifts from 142 to 133 V, and the GMPP voltage shifts from 60 V to 54.5 V. As the temperature changes, the open-circuit voltage, LMPP, and GMPP positions all shift. As temperature rises, all voltages and output power decrease. The temperature between the panels is at the same level, and there may be a slight temperature difference between panels. The total shift of the panel's voltages equals the sum of the shifted voltages of individual panels.

## Simulation results

A simulation model is created in MATLAB Simulink. The model comprises four series-connected solar arrays. Each array comprises two parallel panels, each with a 250-W capacity, as shown in Table 1. The system uses a boost DC/DC converter to supply the electrical load. The proposed MPPT is used to control the boost converter. The performance of the proposed approach was evaluated in comparison to the most well-known MPPT strategies, such as INC, P&O, and PSO.

This comparison considers three variables: the MPPT's ability to extract maximum power from solar panels under normal irradiance, partial shade, and gradually changing irradiance conditions; the fast-tracking time; and the decrease in size of output power steady-state ripples.

**Figure 9:**
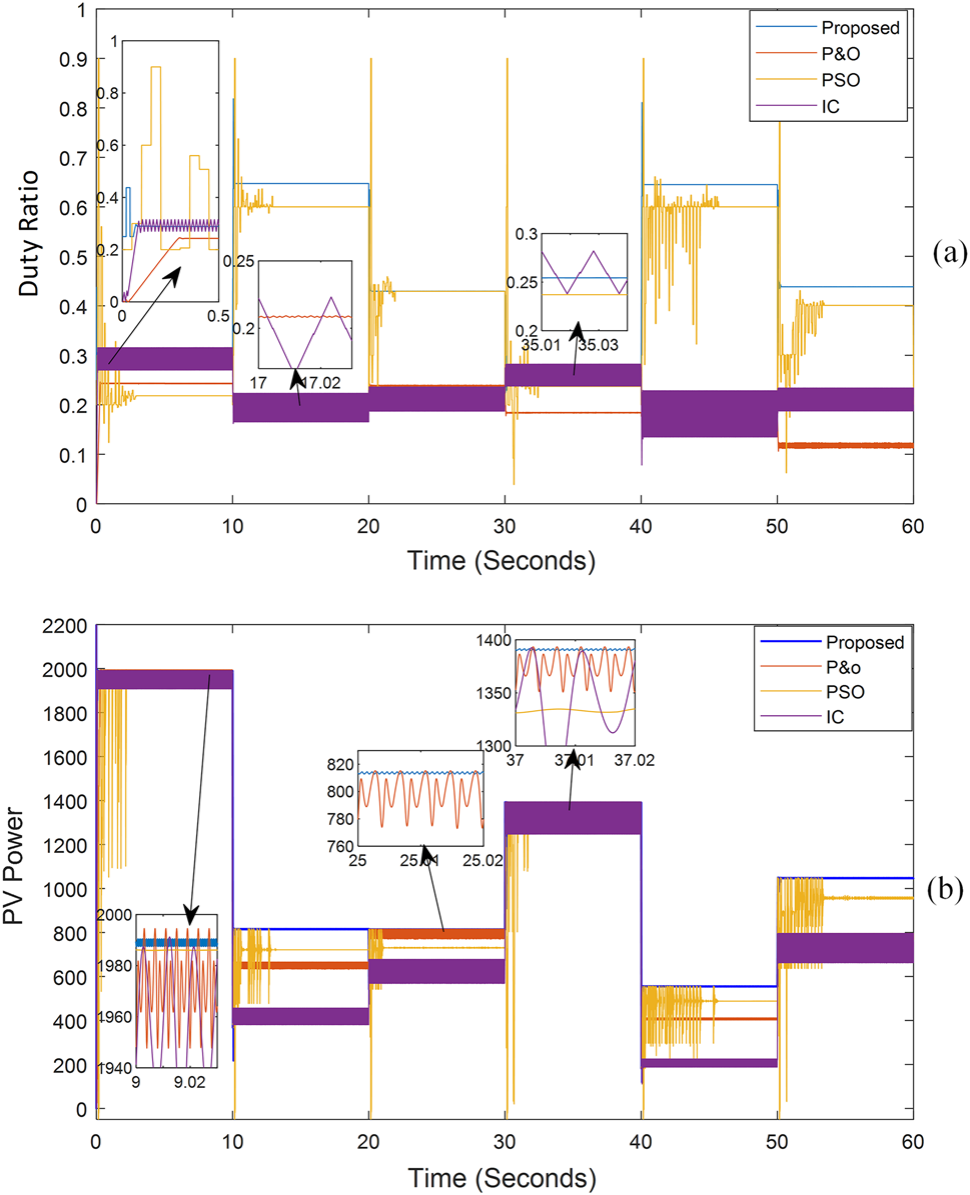
MPPT outputs in case of different shading patterns: (**a**) DC/DC boost converter duty ratio over time, (**b**) PV system output power over time.

The system's output, when exposed to shaded patterns, is shown in Fig. 9. The system was exposed to the irradiance patterns listed in Table 2. In the most challenging scenarios, our proposed method's tracking time did not exceed 0.2 s, and the amount of power change did not exceed 0.3% of the PV power. In contrast to other methods, the proposed approach never missed the global maximum point in any pattern.

**Table 2:** Output power comparison between (INC, P&O, PSO, and proposed technique).

Radiation patterns		MPP	IC	P&O	PSO	Proposed
[1000, 1000, 1000, 1000] t = 0:10 s	P	1989	1954.7	1972.4	1973.3	1989
	∆P%		4.29%	2.4%	0.24%	0.13%
	Tr _t		0.1 s	0.25 s	2.2 s	0.1 s
[200, 400, 800, 1000] T = 10:20 s	P	815	435.9	654.9	717.58	815
	∆P%		17.4%	5.1%	0.13%	0.3%
	Tr _t		0.06 s	0.04 s	2.5 s	0.1 s
[300, 500, 700, 900] T = 20:30 s	P	813.7	644.7	799	730.4	813.63
	∆P%		17.1%	5.8%	1%	0.21%
	Tr _t		0.02 s	0.06 s	1 s	0.12 s
[650, 700, 750, 950] T = 30:40 s	P	1390.9	1335.2	1375.7	1332	1390.8
	∆P%		10.9%	3.3%	0.3%	0.13%
	Tr _t		0.02 s	0.08 s	1.5 s	0.125 s
[100, 250, 550, 650] T = 40:50 s	P	554.8	215.9	408.37	473.2	554.8
	∆P%		17.2%	2.3%	0.1%	0.3%
	Tr _t		0.08 s	0.05 s	4.5 s	0.13 s
[350, 650, 850, 950] T = 50:60 s	P	1047.4	754.65	789.03	945.6	1047.3
	∆P%		17.8%	4.9%	1.2%	0.2%
	Tr _t		0.04 s	0.1 s	3.5 s	0.2 s

Where: Tr_t is the tracking time.

**Figure 10:**
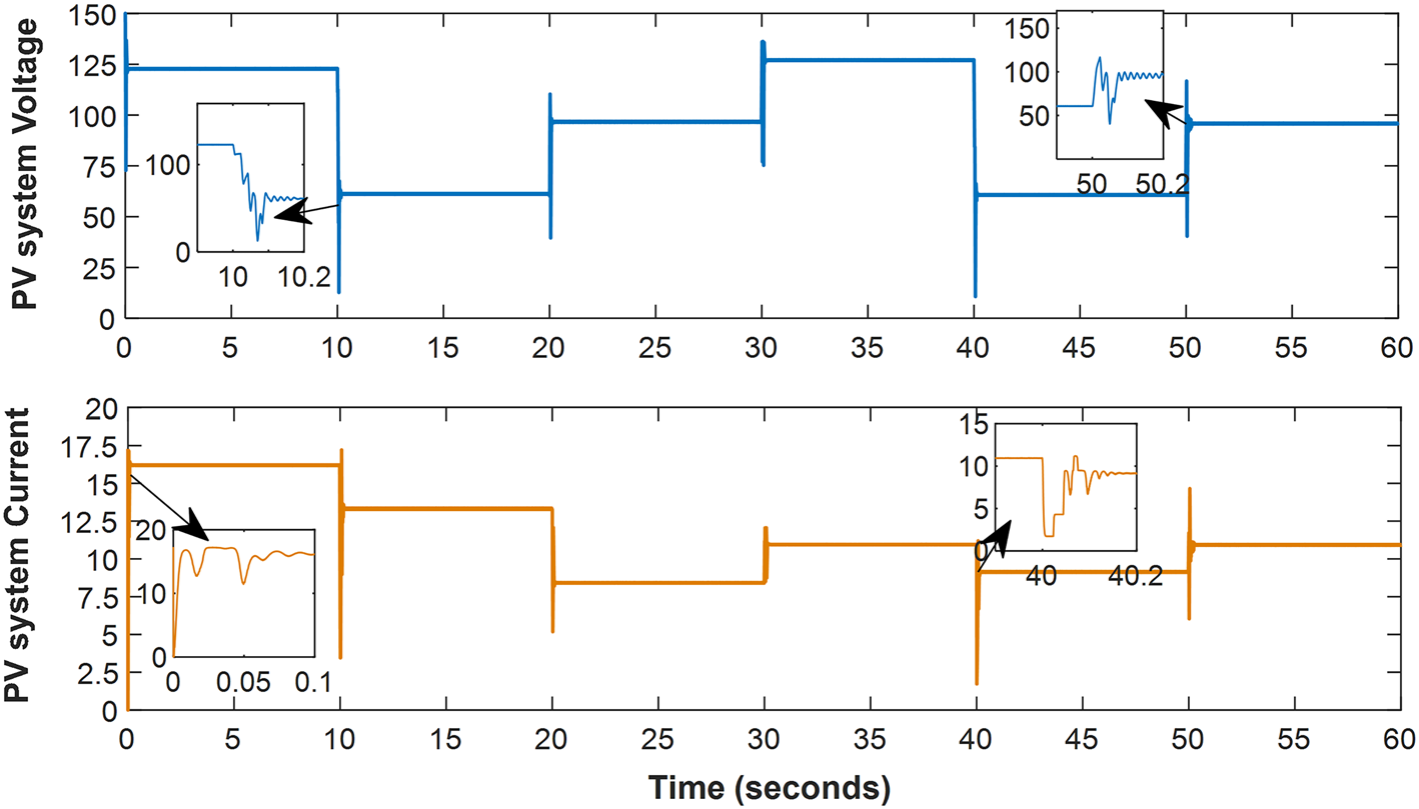
The system current and voltage versus time.

Furthermore, the proposed system was tested on the shaded patterns shown in Fig. 6, following the sequence presented in Table 2, with each test lasting for 10 s. The proposed method's tracking time was observed to be less than 0.15 s, with a few exceptions reaching 0.2 s. This was based on the power curve with time shown in Fig. 9, as well as the voltage and current curves with time displayed in Fig. 10. Notably, neither the voltage nor the current reaches zero, as the system does not need to return to the open circuit point for calibration. Additionally, the ripple in voltage, current, and power is minimal, dropping below 0.3% of the PV power once reaching the MPP. Figures 9 and 10 show the ease and speed of our proposed technique, which employs a global search followed by enhanced INC and proceeds in quick, accurate, and steady steps.

**Figure 11:**
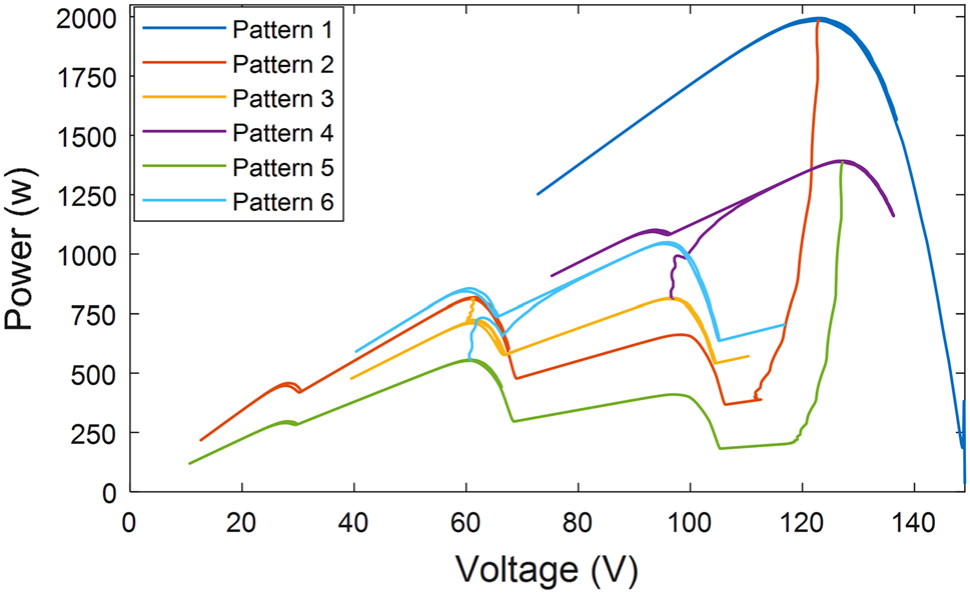
P–V characteristics curve outed from the proposed system.

The system's P–V characteristics are displayed in Fig. 11. As seen in the figure, the system searches the first LMPP near the open circuit voltage in the first scenario where the irradiance is uniform, then moves on to the next nearest one to confirm that the irradiance is uniform, before returning to the first point. Then, at a recorded tracking time no greater than 40 ms, the enhanced INC starts to determine GMPP accurately. The smooth transition of the system is noted when moving to the next pattern at the 10th second, and the system tests the first LMPP, then the second, then the third, and upon reaching the fourth, it returns to the third point because it is near the GMPP. Similarly, in the third pattern at the 20th second, it begins testing the first point until it reaches only the third point, then returns to the second point because it is close to the GMPP.

**Figure 12:**
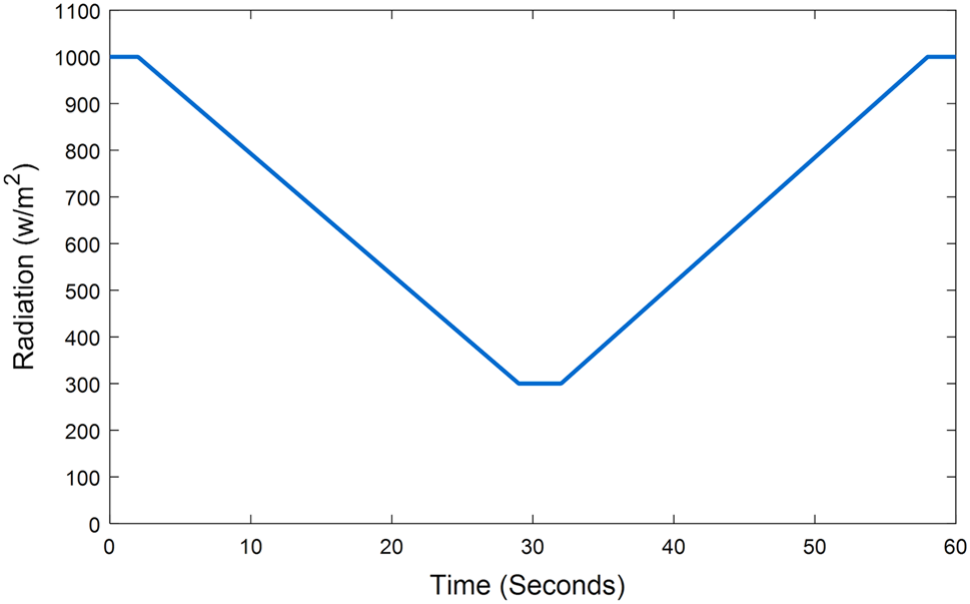
Irradiance of PV system.

The solar irradiance on the panels decreases uniformly from 1000 watts at the 2nd second to 300 watts at the 29th second, then stabilizes at 300 watts for 3 s before increasing once more at the 32nd second and reaching 1000 watts at the 58th second, as shown in Fig. 12.

**Figure 13:**
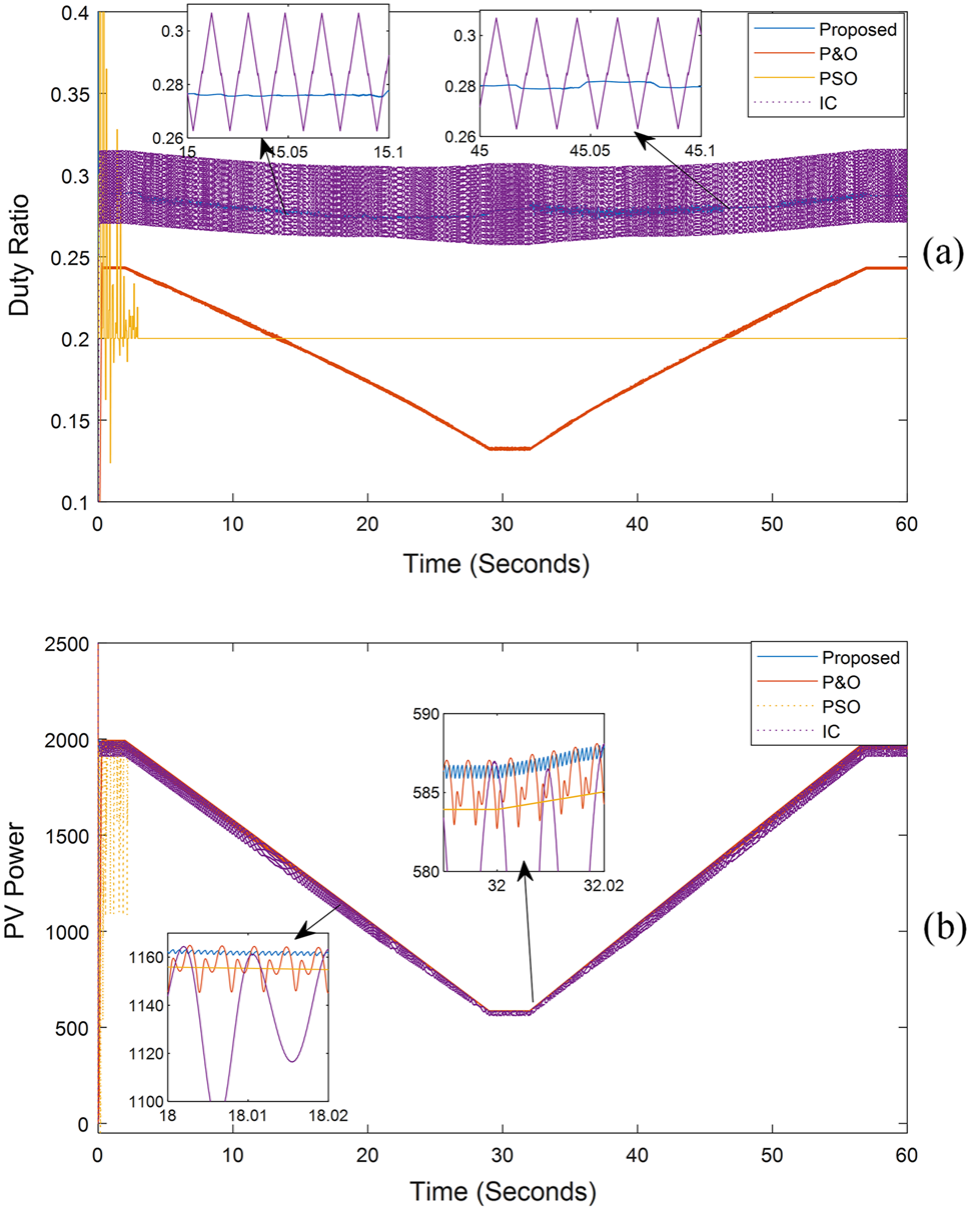
MPPT system output in case of radial decrease and increase of irradiance: (**a**) Duty Ratio, (**b**) Output power.

**Figure 14:**
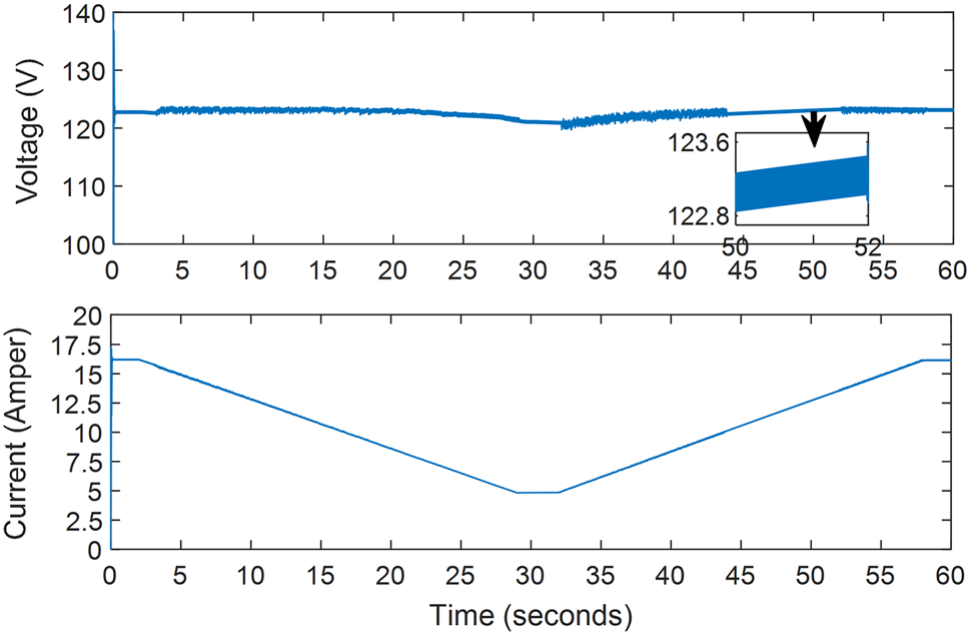
Output voltage and current versus time in case of gradually changing irradiance.

The system's response to this ramping change in irradiance intensity is illustrated in Fig. 13. This figure highlights the proposed technique's quick response time and ability to provide maximum power with minimal ripple compared to other methods. The output of the other techniques was also close to the maximum power due to the uniform irradiance. From Figs. 13 and 14, the system can reach the MPP with continuous, progressive changes in irradiance, whether decreasing or increasing. Figure 14 displays the voltage and current continuously decreasing before stabilizing at the 29th second and then increasing once more before the 32nd second.

**Figure 15:**
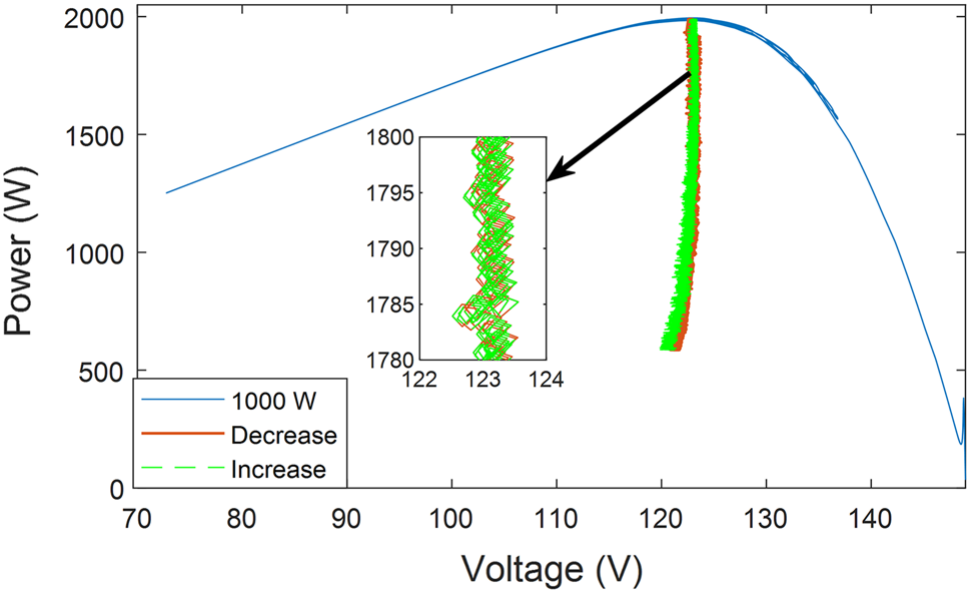
P–V characteristics curve in case of gradual change of irradiance.

The voltage's continuous fluctuation and its rise and fall indicate the system's ability to adjust the voltage to meet the MPP in response to a change in irradiance. Figure 15 illustrates the power changes versus voltage changes as the irradiance level gradually drops from 1000 w/m^2^ to 300 w/m^2^, and how the system adapts its search for the MPP in a tight space with each change. The figure demonstrates that the system can track MPPs accurately, even when the irradiance decreases and then increases again. The proposed tracker is not susceptible to diffusion or selecting the incorrect MPPs, thus ensuring its accuracy.

By comparing the proposed method findings with modern approaches, including^14^, boasting a tracking time of 0.648 s and an efficiency rate of 99.84%,^35^, demonstrating a tracking time of 0.46 s and an efficiency of 99.77%, and^10^, showcasing a tracking time of 1.789 s with an efficiency rate of 99.56%, it becomes apparent that the proposed algorithm delivers superior results. More precisely, the proposed algorithm attains a remarkable tracking time of just 0.2 s while maintaining an exceptional efficiency rate of 99.99%. The proposed control system is distinguished by its simplicity and the minimal degree of fluctuations, which do not surpass 0.3%. These elements set the proposed system apart from existing approaches.

## Conclusion

This paper examines the performance of PV systems under uniform irradiance and partial shading. Although partial shade is not a permanent situation for photovoltaic systems, it can affect the efficiency of solar panels temporarily due to weather conditions or permanently due to damage to certain panel components. A dynamic technique that combines rapid global search with enhanced INC is introduced. Simulation results demonstrate the proposed approach's superior accuracy, fast tracking, and minimal output power ripples compared to other methods. The suggested MPPT provides the following outcomes for PV systems:

- The standard tracking time is less than 0.2 s under all operating conditions.
- The output stability at the MPP shows only a 0.3% variation in PV output power.
- The enhanced INC algorithm achieves the MPP in a shortened period of less than 40 ms.
- A combination of superior power efficiency, enhanced reliability, and reduced implementation costs.

In future work, the proposed system will undergo practical experimentation on a photovoltaic system comprising a substantial number of series-connected modules. A commercial microcontroller will be employed to evaluate the tracking performance, reliability, and efficiency of the system as an economically viable candidate for large-scale PV system applications.

## Data Availability

The datasets used and/or analyzed during the current study available from the corresponding author on reasonable request.

## Abbreviations

PV: Photovoltaic
MPP: Maximum Power Point
MPPT: Maximum PowerPoint Tracker
GMPP: Global Maximum PowerPoint
LMPP: Local Maximum PowerPoint
INC: Incremental Conductance
PSO: Particle Swarm Optimization
P&O: Perturb and observe
HC: Hill Climbing

## List of symbols

${e}^{-}$: Is the electron charge (Coulombe)
V: Is the cell Voltage difference (V)
${m}_{e}$: Is the electron mass (kg)
$v$: Is the electron speed (m/s)
${I}_{L}$: The load current (Ampere)
${I}_{PV}$: PV cell current (Ampere)
${I}_{sh}$: Shunt resistor current (Ampere)
T: PV temperature (k)
K: Boltzmann constant (J/K)
Α: Diode ideality factor
I_PV_: Is the PV current (Ampere)
I_d_: Is the diode current (Ampere)
${R}_{sh}$: Are the shunt losses (ohm)
R_s_: Is the series resistance of the panel (ohm)
${I}_{o}$: Diode saturation current (Ampere)
P: PV output power (W)
V: PV output voltage (V)
I: PV output current (Ampere)
ε: The highest allowable limit of power fluctuation without changing the irradiance and is taken to be [5%:10%]
${V}_{oc}$: PV system open circuit voltage (V)
${V}_{mpp}$: PV system maximum power point voltage (V)
${I}_{mpp}$: PV system maximum power point current (V)
${I}_{pv}$: PV output current (Ampere)
${I}_{sc}$: PV short circuit current (Ampere)
$${V}_{GMPP}$$: GMPP voltage
V(store): The open circuit voltage calculated by the algorithm
M: Number of parallel modules
N: Number of series module
$\gamma$: Is a scaling factor
Tr_t: Tracking time (Seconds)
